# In Vitro and In Vivo Biological Assays of Dextran Coated Iron Oxide Aqueous Magnetic Fluids

**DOI:** 10.3390/pharmaceutics15010177

**Published:** 2023-01-04

**Authors:** Silviu-Adrian Predoi, Simona Liliana Iconaru, Daniela Predoi

**Affiliations:** 1 Département de Physique, École Normale Supérieure Paris-Saclay, 4 Avenue des Sciences, 91190 Gif-sur-Yvette, France; 2 Physique Fondamentale, Université Paris-Saclay, 3 Rue Joliot Curie, 911190 Gif-sur-Yvette, France; 3 National Institute of Materials Physics, Atomistilor Street, No. 405A, 077125 Magurele, Romania

**Keywords:** in vivo, in vitro, dextran, iron oxide, fluid, ultrasound measurement

## Abstract

The iron oxide nanoparticles coated with different surface coatings were studied and characterized by multiple physicochemical and biological methods. The present paper aims at estimating the toxicity in vitro and in vivo of dextran coated iron oxide aqueous magnetic fluids. The in vitro studies were conducted by quantifying the viability of HeLa cells after their incubation with the samples (concentrations of 62.5–125–250–500 μg/mL at different time intervals). The estimation of the toxicity in vivo of administering dextran coated iron oxide aqueous magnetic fluids (DIO-AMF) with hydrodynamic diameter of 25.73 ± 4 nm to Male Brown Norway rats has been made. Different concentrations (62.5–125–250–500 μg/mL) of dextran coated iron oxide aqueous magnetic fluids were administered for 7 consecutive days. Hematology and biochemistry of the Male Brown Norway rats assessment was performed at various time intervals (24–72 h and 21–28 days) after intra-peritoneal injection. The results showed that high concentrations of DIO-AMF (250 and 500 μg/mL) significantly increased white blood cells, red blood cells, hemoglobin and hematocrit compared to the values obtained for the control group (*p* < 0.05). Moreover, following the administration of DIO-AMF, the levels of alkaline phosphatase and aspartate aminotransferase increased compared to the control group (*p* < 0.05). After DIO-AMF administration, no significant difference was observed in the levels of alanine aminotransferase, gamma-glutamyl transpeptidase, urea and creatinine compared to the control group (*p* < 0.05). The results of the present study showed that dextran coated iron oxide aqueous magnetic fluids in concentrations lower than 250 μg/mL are reliable for medical and pharmaceutical applications.

## 1. Introduction

In the last years, the development of engineered particles for use in various biomedical applications has increased due to the progress made in the field of nanotechnology [1,2]. Nowadays, either numerous products based on nanoparticles are already used in biomedical properties, or they are in clinical testing for therapeutic and diagnostic purposes [1]. The potential of inorganic nanoparticles for medical and biomedical applications has been investigated during the years, and studies regarding the nanoparticles’ interactions with cellular systems have reported that, while such interactions could be beneficial both in diagnosis and therapeutics, their use could also result in undesirable toxicity [3,4,5,6,7,8,9,10,11]. Among the most studied inorganic materials, iron oxide nanoparticles have been extensively used in biomedical applications during the last two decades, either as effective bio-imaging contrast agents or for tumors or vascular imaging [4,12]; in drug delivery as carriers of biomolecules, such as drugs, nucleic acids and peptides for controlled delivery to specific organs and tissues [13]; in gene therapy [14]; for in vivo tracking of labeled cells [15]; and as iron supplements for patients with anemia [16]. In order to be used in these applications, the physiochemical and surface properties of the iron oxide nanoparticles need to be carefully designed to consider the synergistic of the body’s innate biological barriers as well as their short- and long-term side effects when administered to the human body. During the years, the study reported that there are several criteria that should be taken into consideration for developing iron oxide nanoparticles for biomedical applications, such as shape, size-distribution, charge, coating molecules and plasma protein [17]. The surface properties of nanoparticles can greatly affect their compatibility in the bloodstream, and some blood constituents have the ability to render nanoparticles and their drug complexes inactive by immunological reactions [18]. There have been studies that reported that nanoparticles interactions with immune cells could lead to their stimulation or suppression [19,20,21]. It was determined that the ability of nanoparticles to induce an immune response could be influenced by particle size, charge and hydrophobicity, surface targeting moieties, therapeutic payload and route of administration [21]. The biodistribution and pharmacokinetics of nanoparticles in different organs have been studied through the years [19,20,21]. Recent advancements in the area of materials science in the synthesis, characterization and surface functionalization of iron oxide nanoparticles have aided researchers in their quest to improve the biodistribution and pharmacokinetics properties of these nanoparticles. Depending on their surface properties, and its chemistry, the expected pharmacokinetic and biodistribution behavior of the iron oxide nanoparticles may considerably differ [22]. Along the years, many types of polymers, such as dextran, chitosan, starch and dextrin [13,23,24], have been used for in situ coating and functionalization of magnetic iron oxide nanoparticles. These coated nanoparticles were investigated, and some of them were successfully translated to clinical trials and then to medical use [25]. In the last years, the U.S. Food and Drug Administration (FDA) [3,26] has approved contrast agents for MRI based on magnetite nanoparticles (Endoremt or Feridex I.V). Dextran is a polysaccharide extensively used in various in vivo applications [27]. For medicinal purposes, dextran is used as an antithrombotic due to its ability to reduce blood viscosity and as a volume expander in hypovolemia [28]. Furthermore, Dextran 70 is considered one of the most important medications needed in a health system by the WHO Model List of Essential Medicines [29]. In this context, dextran coated magnetic nanoparticles have been investigated for biomedical applications [23,30,31]. Studies regarding the pharmacokinetic and toxicity properties of dextran coated magnetic particles revealed these nanomaterials to be sufficiently nontoxic and biodegradable to be suitable for biomedical applications [32]. In 1996, Feridex I.V. (ferumoxides) has been the first nanoparticle-based iron oxide imaging agent FDA approved for the detection of liver lesions. Moreover, another version with smaller particle size, Combidex (ferumoxtran-10), has been used for imaging of prostate cancer lymph-node metastases, and Feraheme (ferumoxytol) has been approved to be used in the treatment of iron deficiency anemia in adult patients with chronic kidney disease. Currently, Ferumoxytol is under clinical investigation for being used in the detection of brain neoplasms, central nervous system inflammation and cerebral metastases [30,33].

In addition, aspects related to the biodistribution of iron oxide nanoparticles coated with different materials have been studied recently [34,35,36,37,38,39]. In their study regarding the “Genotoxicity and biocompatibility of superparamagnetic iron oxide nanoparticles: influence of surface modification on biodistribution, retention, DNA damage and oxidative stress”, Gosh et al. [34] reported the use of poly (lactic-co-glycolic acid) together with either didodeclydimethyl-ammonium-bromide or α-tocopheryl-polyethleneglycol-succinate in order to reduce the cytogenotoxicity and the generation of reactive oxygen species of iron oxide nanoparticles. On the other hand, Bolandparvaz et al. [35], reported that iron oxide nanoparticles functionalized with dextran were distributed in the organism respecting the normal circulation routes and caused no adverse effects. Moreover, Bolandparvaz et al. [35], in their study, highlighted that the exposure to iron oxide nanoparticles functionalized with dextran at a determined dosage did not have any significant toxic effects.

Even though, currently, several dextran-coated iron oxide nanoparticles are in preclinical and clinical trials, there is not enough information available concerning the influence of such particles on cells in culture or about their in vivo behavior. Therefore, the development of new and improved engineered dextran coated iron oxide nanoparticles and complex study regarding their biological properties are of great interest in the medical field.

In vivo studies are very important because in vitro studies cannot provide significant information on the response of the physiological system. Thus, the in vivo study of the toxicity of magnetic iron oxide nanoparticles is very useful especially since these studies are quite limited compared to the wide field of applicability. As there is little research on hematological and biochemical analyses, in this study, we aimed to bring results on the in vivo toxicity of dextran coated iron oxide aqueous magnetic fluids with a hydrodynamic diameter of 25.73 ± 4 nm for 28 days. The dextran coated iron oxide aqueous magnetic fluids were injected intraperitoneal in different concentrations (62.5–500 μg/mL) for seven consecutive days, and the hematological and biochemical analyses were performed at different time intervals (24 h, 72 h, 21 days and 28 days). The stability studies on dextran coated iron oxide aqueous magnetic fluids using ultrasound measurement presented in this study is a novelty and a great progress regarding the evaluation of the stability of concentrated magnetic fluids. The results of this in vivo research will evaluate which concentration of dextran coated iron oxide aqueous magnetic fluids has no toxic effect and would be suitable for various applications.

## 2. Materials and Methods

### 2.1. Sample Preparation

#### 2.1.1. Materials

Ferric chloride hexahydrate (FeCl_3_⋅6H_2_O), ferrous chloride tetrahydrate (FeCl_2_⋅4H_2_O), sodium nitrate (NaNO_3_), natrium hydroxide (NaOH), perchloric acid (HClO_4_) and chlorhidric acid (HCl) were purchased from Merck. Double distilled water and deionized water were used in the synthesis and for rinsing the clusters.

#### 2.1.2. Synthesis of Dextran Coated Iron Oxide Aqueous Magnetic Fluids

In order to synthesize the iron oxide aqueous magnetic fluids (IO-AMF) the ferric chloride hexahydratate (FeCl_3_ × 6H_2_O) and ferrous chloride tetrahydrate (FeCl_2_ × 4H_2_O) in 2 M HCl were combined at room temperature and used in agreement with previous reported papers [40,41,42,43]. The ratio of Fe^2+^/Fe^3+^ was ½. The mixture of Fe^2+^ and Fe^3+^ and a solution of NaNO_3_ (1 mol/L^−1^) was added drop by drop into a NaOH (2 mol⋅L^−1^) solution for 60 min under vigorous stirring. The immediately formed black precipitate of magnetite immediately was transformed into maghemite by adding into maghemite particles after repeated treatment with HClO_4_ (3 mol⋅L^−1^) solution until the ratio of Fe^2+^ /Fe^3+^ in the bulk sample was 0.05, approximately [43]. After the latest treatment, the aqueous magnetic fluids were separated by centrifugation (10,000 rpm) and then washed in double distilled water and dispersed in deionized water. Functionalized iron oxide nanoparticles were generated by mixing the iron oxide aqueous magnetic fluids with dextran solution (10 g in 100 mL of water) at a defined mass ratio [IO-AMF] to [dextran] of 1:2. The mixture was sonicated for 1 h and then further stirred for 12 h. The solution containing dextran coated IO-AMF was washed by means of magnetic columns. The iron content of final dextran coated iron oxide aqueous magnetic fluids (DIO-AMF) was 0.38 mol/L [44].

### 2.2. Characterizations

#### Characterization of Dextran Coated Iron Oxide Aqueous Magnetic Fluids

The morphology of dextran coated iron oxide aqueous magnetic fluids was examined by transmission electron microscopy (TEM) measurements using a CM 20 (Philips-FEI, Hillsboro, OR, USA) transmission electron microscope equipped with a Lab6 filament operating at 200 kV.

The X-ray diffraction analysis for dextran coated iron oxide aqueous magnetic fluids was performed using a Bruker D8 Advance diffractometer (Bruker, Billerica, MA, USA) with nickel filtered Cu K_α_ (λ = 1.5418 Å) radiation and a high efficiency one-dimensional detector Lynx Eye type (Bruker, Billerica, MA, USA) operated in integration mode. The diffraction patterns have been recorded in the 2θ range 20–70°, with a step of 0.02° and 34 s measuring time per step.

The mean size of DIO-AMF particles was determined by mathematical Scherrer [45,46] equation:

D = Kλ/β Cos θ (1)

where K is the shape factor which usually takes a value of about 0.9; λ is the X-ray wavelength; β is the line broadening at half the maximum intensity (FWHM); and θ is the diffraction angle.

Using the ultrasound measurements (US), we evaluated the stability of concentrated dextran coated iron oxide aqueous magnetic fluids resulting from the synthesis process. The ultrasound measurement was effectuated in agreement with anterior studies [47]. For the correctness of the stability evaluation, double distilled water was taken as reference fluid. It is well known that double distilled water is the most stable fluid. The double distilled water was measured in the same experimental conditions as the aqueous magnetic fluid.

### 2.3. Biological Studies

#### 2.3.1. Quantitative Cell Viability Assay

The biocompatibility of the DIO-AMF was assessed by quantifying the viability of HeLa cells after their incubation with the samples. For this purpose, the DIO-AMF were diluted to concentrations of 62.5, 125, 250 and 500 μg/mL in 96-well culture plates (Sarstedt, Nümbrecht, Germany) in DMEM medium supplemented with 10% FCS and 100 μg/mL penicillin/streptomycin. The culture plates were then filled with 1 × 10^4^ HeLa cells. The HeLa cells culture without DIO-AMF was chosen as control. The HeLa cells viability after incubation with DIO-AMF was assessed at different time intervals. For this purpose, after 24 h, 72 h and 7 days of incubation, the medium was removed and the cells washed with BS and stained with fluorescein diacetate (FDA; Thermo Fisher, Waltham, MA, USA). The fluorescence was quantified at 535 nm using a microplate reader (Perkin Elmer, Waltham, MA, USA), and the values were normalized, taking into account the control culture [48].

#### 2.3.2. Animals Experimental Design

The in vivo studies were conducted on Male Brown Norway rats (weighing 200 g, 10 weeks age) provided by the National Institute of Research and Development for Microbiology and Immunology “Cantacuzino”, Bucharest. The Male Brown Norway rats were housed in polycarbonate cages in controlled environmental conditions at an ambient temperature of 22 ± 2 °C for 12 h light/dark cycles and a humidity of 60 ± 10%. For acclimatization in controlled environmental conditions, the rats were kept in the laboratory one week. All the experiments on rats were realized under specific pathogen-free conditions in concordance with the International Animal Care Policies and the NIH Guide for the Care and Use of Laboratory Animals. In the present study, 35 Male Brown Norway rats were randomly divided into 5 groups of 7. Of the five groups, one was the control group. The other four groups were the experimental groups. A group of seven mice was assigned to each dose of injected DIO-AMF nanoparticles. The administration of DIO-AMF nanoparticles was effectuated using intraperitoneal injection. The rats of groups 2, 3, 4, and 5 received 1 mL of DIO-AMF at doses of 62.5 μg/mL 1, 125 μg/mL, 250 μg/mL and 500 μg/mL for 7 consecutive days. At the end of 28 days, all rats in the 5 groups were starved overnight. After that, the animals were anesthetized with ketamine 100 mg/kg (Rotexmedica Co., Bunsenstrasse, Germany) and xylazine 10 mg/kg (Alfasan Co., Kuipersweg, The Netherlands), and hematological and biochemical samples were taken for laboratory analysis.

#### 2.3.3. Hematological and Biochemical Analysis

In order to conduct the hematological and biochemical analysis, the blood samples were collected by intra-cardiac puncture. The blood was directly collected in EDTA-coated vials for hematological analysis. An automated hematological analyzer (Sysmex Cell Counter Model K-1000) was used to determine the hematologic toxicity. The samples were examined in the first 2 h after harvest. During this time the tubes were stored in the refrigerator at a temperature of around 8 °C. The hematological parameters evaluated in this study were hemoglobin (Hb) levels, mean corpuscular hemoglobin (MCH), mean corpuscular hemoglobin concentration (MCHC), mean corpuscular volume (MCV), blood cells (WBC), red blood cells (RBC), hematocrit (HCT) and platelet count (PLT). In the same time, for the serum biochemical analysis, the blood was collected in a clotted vial. The whole blood was centrifuged at 3000 rpm for 15 min to obtain the serum using an automated analyzer (Hitachi 912 Chemistry Analyzer). Liver function was assessed on the basis of alanine aminotransferase (ALT), aspartate aminotransferase (AST), γ-glutamyl transpeptidase (GGT) and alkaline phosphatase (ALP). Urea (UREA) and creatinine (CREA) were examined to assess nephrotoxicity. The level of biochemical parameters was measured using a photometric method using quantitative diagnostic kits.

### 2.4. Statistical Analyses

All in vitro and in vivo experiments were effectuated in triplicate. Moreover, all measurements were performed three times. Data were plotted as mean ± standard deviation (SD).

## 3. Results and Discussions

**Figure 1:**
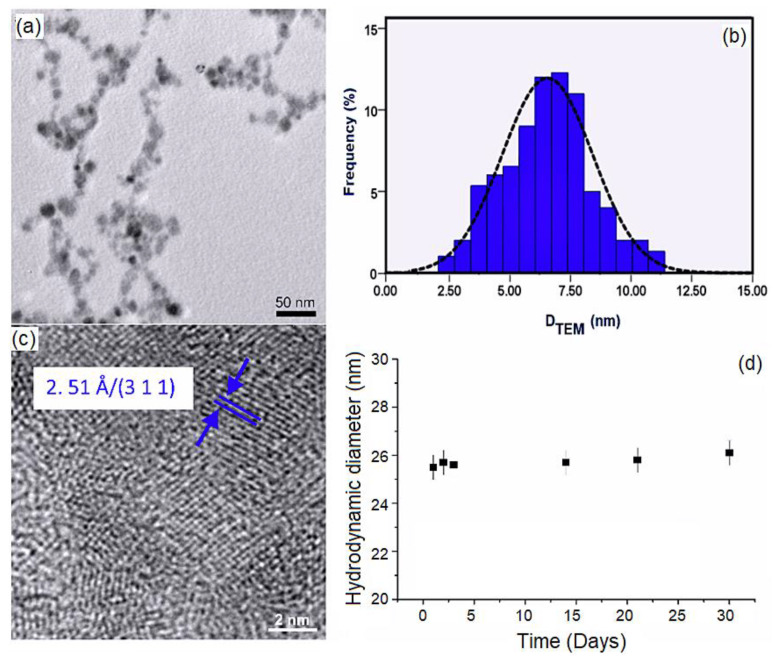
TEM image (**a**), size distribution histogram (**b**), HRTEM image (**c**) and average hydrodynamic diameter of the DIO-AMF nanoparticles as a function of time (**d**).

In order to evaluate the morphology of DIO-AMF particles, a drop of the magnetic suspension was deposited on copper grids and dried at room temperature. The TEM observation of DIO-AMF was presented in Figure 1. Figure 1a revealed the TEM image of DIO-AMF at low magnification. The photomicrographs of TEM showed the spherical shape and a narrow size distribution of the DIO-AMF particles. A good dispersion of the particles was also observed. The histogram, resulting from measuring the diameter of a number of approximately 700 particles in TEM images, gives us an overview of the population distribution of DIO-AMF nanoparticle core diameters. According to the size distributions (Figure 1b), the average particle size of DIO-AMF was D_TEM_ = 6.8 ± 1 nm. Additionally, HRTEM images presented in Figure 1c provide the confirmation that dextran coating of the IO-AMF nanoparticles was successfully achieved.

To determine the hydrodynamic diameter (D_H_) of the DIO-AMF nanoparticles, the dynamic light scattering (DLS) was used. The DLS measurement provides information on the particle size with the included coating as opposed to TEM, which provides only core information (Figure 1d).

**Figure 2:**
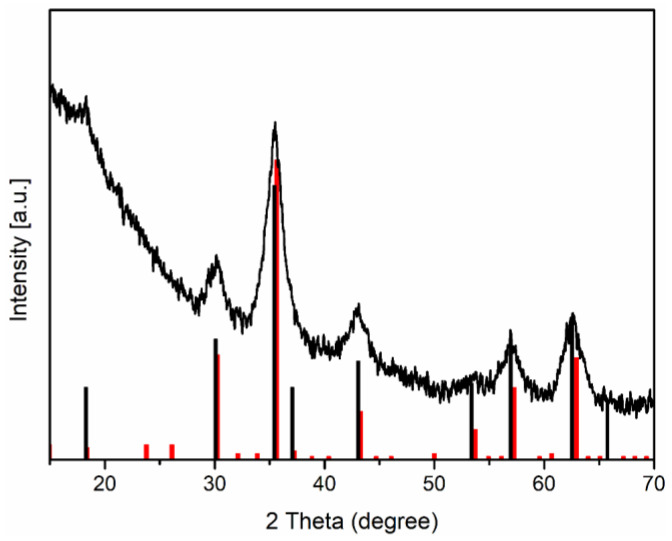
XRD patterns of dextran coated iron magnetic nanoparticles and reference patterns for (black) Fe_3_O_4_ PDF #65-3107 and (red) γ-Fe_2_O_3_ PDF # 25-1402.

The hydrodynamic diameter of the DIO-AMF nanoparticles was measured at various time intervals over 30 days. Figure 1d shows the hydrodynamic diameter of DIO-AMF nanoparticles as a function of time. It can be seen that DIO-AMF nanoparticles showed good stability over a period of 30 days. The average hydrodynamic diameter of DIO-AMF nanoparticles was D_H_ = 25.73 ± 4 nm. The DLS technique was used to estimate the size of nanoparticles in suspension in all types of solvent. DLS measures a relaxation time for the decay of the autocorrelation function of the scattered light from which a diffusion coefficient inversely proportional to the particle size can be extracted. On the other hand, TEM shows an image of an area of the sample. Thus, DLS provides information about the hydrodynamic size, which is the size of the nanoparticle plus the liquid layer around the particle, while the size measured by TEM provides the actual size of the nanoparticle (the core of the particle which is magnetic, in this case). The DH/DTEM ratio was equal to 3.78, which highlights the presence of dextran on the surface of the particles. The value obtained for D_H_ of nanoparticles using DLS measurements was in agreement with previous studies [49,50]. It can be said that the proposed method for abstaining of dextran coated iron oxide aqueous magnetic fluids nanoparticles was efficient. The crystal structures of DIO-AMF were examined by XRD. To investigate the crystal structure of DIO-AMF, the suspension was centrifuged at 12,000 rpm. The resulting powder was dried for 12 h in an oven at 80 °C. The XRD patterns of DIO-AMF sample and the reference peaks for magnetite and maghemite was presented in Figure 2 for comparison. The diffraction characteristic peaks were identified in the XRD pattern at 2θ of 23.79, 30.28, 42, 53.4, 55 and 62.7 associated to their Miller indices (hkl) of (210), (220), (310), (321), (400), (421), (422), (511) and (440) and are in agreement with the cubic structure (Fd3m3 space group) of maghemite (JCPDS card No. 39-1346). The calculated lattice parameter of the obtained DIO-AMF was 8.338 Å. W. Kim and others [51] mentioned that the identification of the phases of magnetite and maghemite are difficult to quantify when they coexist, as they are very close. However, after determining the lattice parameter, we can tell if the identified phase is magnetite or maghemite. For our sample (DIO-AMF), the value of the lattice parameter has a value approximately equal to that for the bulk lattice parameter of γ-Fe_3_O_4_ (a = 8.3474 Å), which allows us to say that the only phase identified was that of the maghemite. The results obtained are in agreement with those presented in previous studies [52,53,54]. The calculated crystallite size (D_XRD_) using the Scherrer’s equation [55] of DIO-AMF nanoparticles was 6.2 ± 0.5 nm. The very good crystallinity of the DIO-AMF nanoparticles was confirmed, which shows that the broadening of the diffraction peaks was due to the nano-sized material. The XRD results were in good agreement with the TEM analysis. The slightly smaller size obtained by XRD compared to that obtained by TEM is due to the measurement techniques.

**Figure 3:**
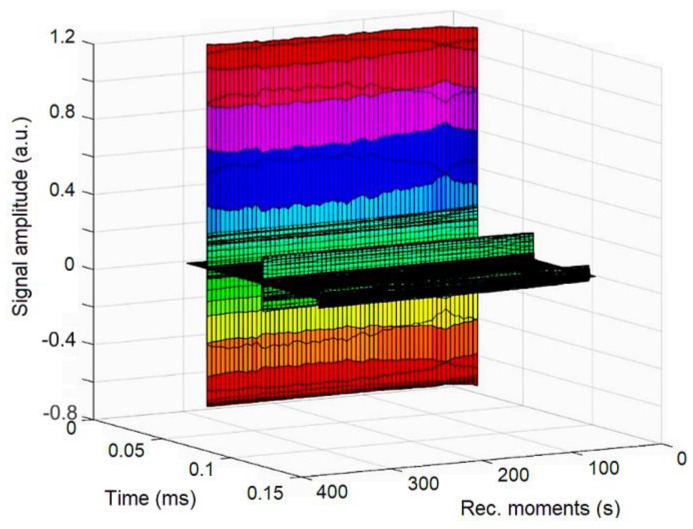
Recorded signals at 5 s recording interval. The amplitudes of the first echo are slowly increasing (in colors). The second (green) and third (black) echo are significantly weaker.

In order to evaluate the stability of concentrated DIO-AMF, ultrasound measurement was conducted. 100 mL of DIO-AMF in the specialized transparent recipient was homogenized for 5 min at room temperature in an ultrasonic bath. After homogenization the ultrasonic pulses were sent through the DIO-AMF. In Figure 3, the signals—recorded at an interval of 5 min from the oscilloscope to a digital storage device—are presented. By studying the evolution of signals over time we can obtain significant information about the stability of the suspension and attenuation vs. time.

**Figure 4:**
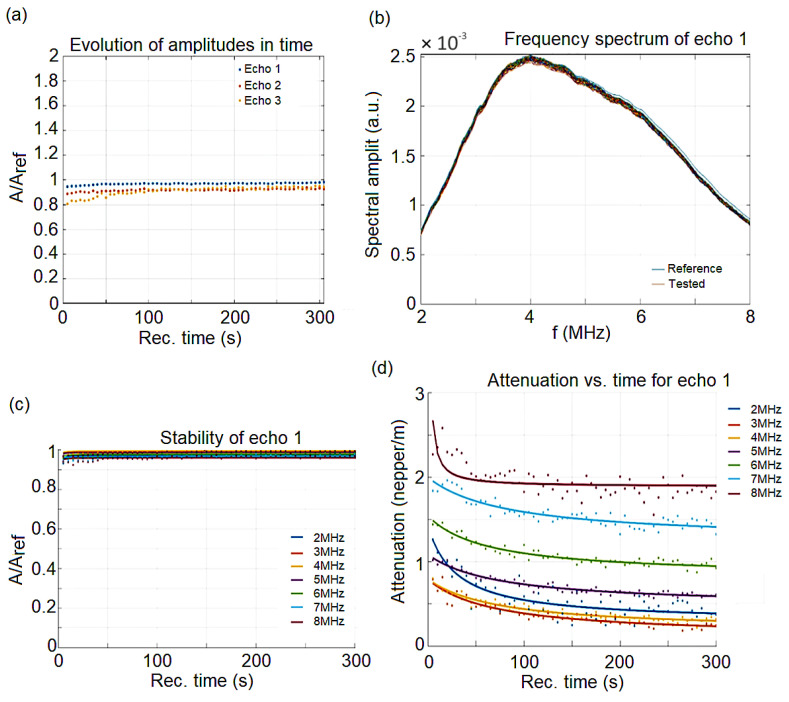
(**a**) Relative amplitudes evolution vs. the recording moments; (**b**) Frequency spectrum of the first transmitted echo. Reference fluid (-); (**c**) Spectral amplitudes relative variation vs. time, for the first echo; (**d**) Attenuation vs. time for the spectral components of the first echo.

The double distilled water was taken as the reference fluid, being measured under the same experimental conditions as the analyzed sample. The processing of DIO-AMF signals was obtained by comparison with the properties of double distilled water. The result is c = 1493.96 m/s, compared to the velocity in the reference fluid c_0_ = 1490.66 m/s at 22.8 °C, but the signal’s velocity through the sample has negligible variations in time so that velocity cannot be used to characterize the sample. The maximum amplitudes of transmitted signals vs. recording moments show clearly the constant amplitudes of all three recorded signals. The signal analysis code shows for the first echo, which is measured with highest accuracy, that the slope of this amplitude vs. time, which is related to the stability parameter, is in this case $s=\frac{1}{A_{m}}\left|\frac{dA}{dt}\right|=0.0027\left(1/s\right)$ with *A_m_* the averaged amplitude of the signals. The value obtained from the calculations shows a good stability, compared to the perfect stability (s = 0). The frequency spectrum of the first transmitted echo represented in Figure 4b is another important characteristic of the suspension. The stability can be followed also on this figure, covering 300 s of recorded signals, since the samples, spectra are almost superposed. This information is confirmed by the variation of spectral amplitudes during the experiment. On Figure 4c is shown the constant amplitude ratios close to 0.93 during the entire experiment and for all tested frequencies. These relative amplitudes allow the computation of the signal’s attenuation. For each spectral component, the attenuation depends slowly on the recording moment during the experiment. For this sample (Figure 4d), there is an initially faster reduction of attenuation for each spectral amplitude, followed after 50 s by a steady value for the 8 MHz component or a slow decrease for the other frequencies.

The cytotoxicity of the DIO-AMF samples was assessed using HeLa cells, which is the first immortal human cell line, known for being durable and prolific [56]. The toxicity of the DIO-AMF was investigated at various time intervals and for different concentrations on HeLa cells.

**Figure 5:**
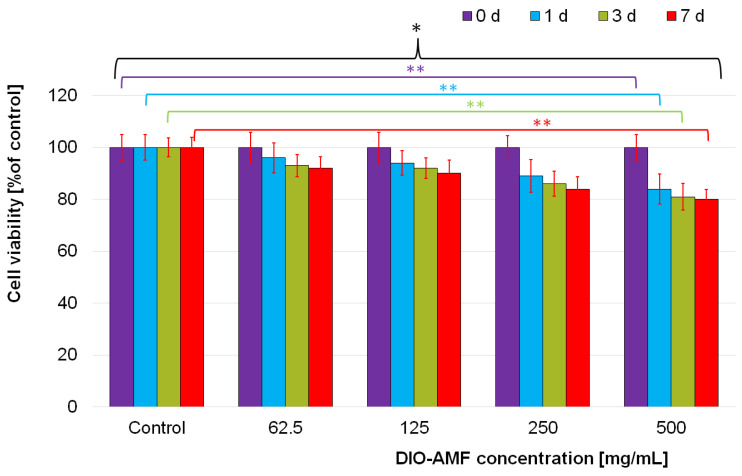
Graphical representation of the time dependent changes of HeLa cell viability in response to DIO-AMF. Cells incubated with 62.5, 125, 250 and 500 μg/mL DIO-AMF for 0, 24 h, 72 h and 7 days. Cell viability analyzed by FDA staining and normalized to untreated control cells. The results are presented as mean ± SD. Statistical analysis was performed by one-way ANOVA. The *p*-values indicated are the following: * *p* ≤ 0.05, ** *p* ≤ 0.001.

Furthermore, the cytotoxicity of the DIO-AMF suspensions was investigated quantitatively by determining the HeLa cell viability incubated with 62.5, 125, 250 and 500 μg/mL DIO-AMF at different time intervals. The results of the quantitative cell viability assay are presented in Figure 5. The cell viability assays revealed that for the HeLa cells incubated with 62.5 and 125 μg/mL DIO-AMF suspensions, at all tested time intervals, there were no representative differences in the values of cell viabilities compared to the control cell culture and to the cell culture at 0 d. The results of the cell viability assay have emphasized that, after 72 h of incubation, the cell viability of HeLa cells decreased from 96% for the cells incubated with 62.5 mg/mL DIO-AMF to 93% for 125 μg/mL, 89% for 250 μg/mL and to 82% for the cell culture incubated with a 500 mg/mL DIO-AMF suspension. A similar behavior was evidenced for the other tested intervals.

The cell viability decreased with the increase of the DIO-AMF suspensions concentration. In addition, the results have also emphasized that the cell viability was influenced by the incubation time. The cell viability values of the HeLa cells incubated with various concentration of DIO-AMF at different time highlighted that the tested suspensions did not present a high toxicity towards HeLa cells and that their toxicity was strongly influenced both by the DIO-AMF concentration as well as the incubation time interval. Moreover, the results showed an almost constant cell density with only moderate declines of cell numbers for all tested concentrations at the tested time intervals.

**Figure 6:**
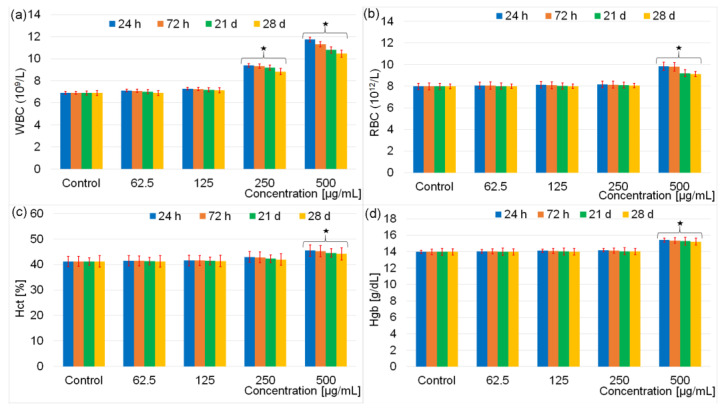
Hematological parameters for the rats exposed to different dose containing DIO-AMF at different time intervals. White blood cells (WBC) (**a**), red blood cells (RBC) (**b**), hematocrit (Hct) (**c**) and hemoglobin (Hgb) (**d**). Represents important difference from the control group (* *p* < 0.05).

In vitro studies, despite the very valuable data they give, cannot provide information on the response of the physiological system. In order to have an objective evaluation of the toxicity of dextran coated iron oxide aqueous magnetic fluids, the results of hematological and biochemical analyses obtained following the intraperitoneal injection of male Brown Norway rats at various time intervals with different concentrations are presented below. Administration of DIO-AMF at various doses (62.5 μg/mL, 125 μg/mL, 250 μg/mL and 500 μg/mL) for a period of 7 consecutive days did not produce clinical signs in mice from the 4 study groups. All 35 Male Brown Norway rats in the 5 study groups survived to the end of the experiment. Moreover, the behavior of the animals remained normal. During the experiments, none of the mice showed fever, weight loss, lethargy, dehydration or ruffled fur. All 35 Male Brown Norway rats were active and nonaggressive during the interactions with other rats from the cage. An important step in assessing the toxicity of DIO-MFA is to analyze hematological parameters. In this study, the analyses performed allowed the evaluation of white blood cells (WBC), red blood cells (RBC), hematocrit (HCT) and hemoglobin (Hgb). The first analysis was performed 24 h after the last injection, and the second analysis 72 h after the injection. The following tests were performed on days 21 and 28 after the injection. The hematological analysis revealing the toxic effect of DIO-AMF after intraperitoneal injection of the rats with different doses at various time intervals is presented in Figure 6.

**Figure 7:**
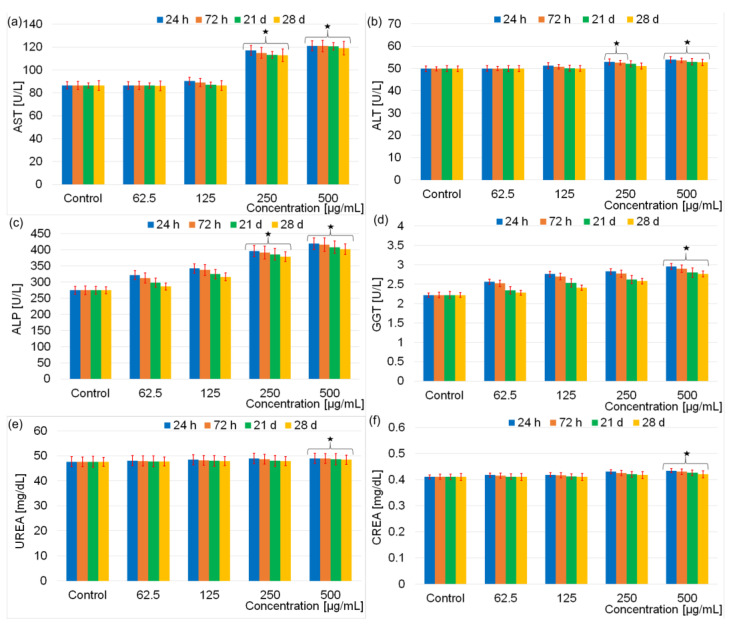
The biochemical results for rats exposed to different doses (62.5–500 μg/mL) containing DIO-AMF at different time intervals as follows aspartate transaminase (AST) (**a**), alanine transaminase (ALT) (**b**), alkaline phosphatase (ALP) (**c**), γ-glutamyl transpeptidase (GGT) (**d**), urea (UREA) (**e**) creatinine (CREA) (**f**). (* *p* < 0.05).

The result of hematology analysis (Figure 6) revealed that there were no effects on the evaluated parameters for the rats exposed to 62.5 μg/mL and 125 μg/mL doses containing DIO-AMF related to the control after 28 days. For the rats exposed to 250 μg/mL dose containing DIO-AMF, the parameters were increased, but no considerable difference was observed. It was observed that WBC increased at DIO-AMF doses of 250 and 500 μg/mL, respectively. RBC values were not influenced following the exposure of rats to doses between 62–250 μg/mL. A slight increase was observed after increasing the concentration to 500 μg/mL. Regarding the Hct values, a slight increase was highlighted following the exposure of the rats to the concentration of 500 μg/mL. On the other hand, Hgb values remained practically unchanged for rats exposed to doses between 62–250 μg/mL and increased in the case of exposure to doses of 500 μg/mL. Comparing the results of the red blood cell investigation tests, we can conclude that the number of red blood cells was most influenced by the increase in concentration. The hematocrit (which represents the size of the volume occupied by the red blood cells in a blood sample in relation to the volume of the sample, expressed as a percentage) was less influenced. The evolution of Hgb was similar to that of Hct, their values being less influenced by the increase in concentration than the total number of red blood cells. Regarding the WBC analysis, the changes observed from 250 μg/mL could suggest a reactivity of the body by increasing the number of antibodies. In conclusion, we could say that the concentration influenced the behavior of white blood cells more than that of red blood cells. To evaluate the biochemical effects of different doses of DIO-AMF (62.5 μg/mL, 125 μg/mL, 250 μg/mL and 500 μg/mL) on rats treated for 7 consecutive days, both liver function analyses (AST, ALT, ALP, GGT) and nephrotoxicity studies (UREA and CREA) were performed. The results of the investigation of the biochemical effects of different doses of DIO-AMF administered to rats for 7 consecutive days are presented in Figure 7.

The tests on aspartate aminotransferase, alanine aminotransferase, alkaline phosphatase, γ-glutamyl transpeptidase, urea and creatinine have provided precious information. It was established that the level of AST and ALP were significant. The increase in AST values (enzyme especially specific to myocardial tissue) at concentrations of 250 and 500 μg/mL suggests a possible damage to the myocardial tissue, which could lead to various myocardial diseases. ALT (liver tissue-specific enzyme) did not show changes when rats were exposed to concentrations of 62.5 and 125 μg/mL of DIO-AMF. This behavior suggests that the liver tissue was not affected at these concentrations of DIO-AMF. At concentrations of 250 μg/mL and 500 μg/mL, a slight increase in ALT values was observed after 24 h. After 72 h, a decrease in ALT compared to 24 h was observed. Moreover, after 28 days, the ALT value is comparable to that of the control, which suggests that the mildly affected liver tissue recovers after 28 days. Alkaline phosphatase (ALP), the specific enzyme mainly for liver and bone tissues, showed variations starting from 62 μg/mL. Taking into account the fact that no changes were observed in the ALT test at concentrations of 62.5 and 125 μg/mL of DIO-AMF and that the slight changes that appear at concentrations of 50 μg/mL and 500 μg/mL after 24 h of incubation return to normal values after 28 days, we could say that, after the exposure of the rats to DIO-AMF, it could be possible to damage the bone tissue. The statement that DIO-AMF could induce bone tissue damage is based on the one hand on the fact that, after the ALT test, increased values were observed even for the lowest concentration, and on the other hand, the ALP values continue to increase for the concentrations bigger. On the other hand, the AST values do not return to values comparable to those for the control even after 28 days. The slight changes observed in the GGT values (enzyme specific to the liver tissue but also to the bile ducts) suggest that there could be a minor influence on the extrahepatic (biliary) pathways. Regarding the evaluation of nephrotoxicity tests (UREA and CREA), the absence of DIO-AMF influence was observed in relation to the control. As a result, it can be said that DIO-AMF does not show nephrotoxicity for the analyzed concentrations.

The aim of this study was to evaluate the in vitro and in vivo toxicity effect of different doses (62.5–500 μg/mL) containing DIO-AMF. For this purpose, the in vitro evaluation of toxicity effect of different doses (62.5–500 μg/mL) containing DIO-AMF at various time intervals on HeLa cells was very useful. On the other hand, an important role in assessing the toxicity effect of DIO-AMF was played by in vivo tests performed on rats. The results of the present study indicated that, at doses greater than 125 μg/mL containing DIO-AMF, a toxic effect was observed in both in vivo and in vitro studies.

The results presented in this study are in good agreement with previous studies regarding the toxicity of various iron oxide nanoparticles [57,58,59,60,61,62,63]. Usually, the different types of cytotoxicity assays and the use of different culture media can also provide variations for the in vitro toxicity results [62]. Studies regarding the toxicity of iron oxide nanoparticles reported a dose dependent effect of the nanoparticles for different cell types. Most often, the cytotoxicity of such particles appears at concentrations higher than 300 μg/mL and for prolonged exposure time [64,65]. Moreover, studies emphasized that the properties of nanoparticles are known to be size and shape dependent, and in consequence, the effects that they could induce to cells and tissues could vary [63,66]. In their study regarding the “Rod-shaped iron oxide nanoparticles are more toxic than sphere-shaped nanoparticles to murine macrophage cells”, the authors showed that rod-shaped nanoparticles were more toxic than the sphere-shaped ones, demonstrating that the shape of the particles has a strong influence on their toxicity [67]. The impact of numerous physicochemical properties of iron oxide nanoparticles on their in vitro toxicity increases the probability of obtaining different results. Therefore, a proper characterization of particles must be presented in order to attest for the variations in the in vitro studies. During recent years, in order to lower the toxicity of iron oxide nanoparticles, various materials were used as coating for iron oxide nanoparticles to stabilize their physicochemical and biological properties [53,54,55,56,57,58,59,60,61,64]. Dextran, silica and PEG coatings have been reported to help lower the toxicity of iron oxide nanoparticles [62,68,69].

Hematological tests have shown that white blood cells are susceptible to physiological responses. Previous studies on the in vivo quantum-dot toxicity assessment [64] have suggested that the increase in white blood cells in rats treated with nanometer-sized semiconductor particles may be recognized as an inflammatory response. In agreement with the studies regarding size-dependent in vivo toxicity of PEG-coated gold nanoparticles [65], all the changes that take place in the red blood cells can be associated with the hematopoietic system. As it can be seen, the studies conducted on rats treated with 500 μg/mL for 7 consecutive days of DIO-AMF suggested that the nanoparticles with a hydrodynamic diameter D_H_ = 25.73 ± 4 nm affect the hematopoietic system. More of that, the DIO-AMF with hydrodynamic diameter of around 25.73 nm can increase or decrease the function of the immune system when injected in high doses (250 and 500 μg/mL) [70,71,72]. In addition, the related hematological factors, including the number of cells in the blood can be modified [70,72].

The results of the present study showed that the injection of DIO-AMF in rats with doses of 62.5, 125, 250 and 500 μg/mL suggests both a possible damage to the myocardial tissue and a possible damage to the bone tissue. Furthermore, a minor influence on the biliary tract could occur.

Our studies of DIO-AMF cytotoxicity are in agreement with previous studies. Moreover, following studies on biocompatibility of magnetite nanoparticles evaluated by in vitro cytotoxicity assays using normal, glia and breast cancer cells have shown that the toxicity of magnetite nanoparticles coated with a bipolar surfactant has not been observed for concentrations in the range of 0.1–10 μg/mL [73]. Another study on “copper oxide nanoparticles are highly toxic: a comparison between metal oxide nanoparticles and carbon nanotubes” [74] demonstrated that the iron oxide nanoparticles were non-cytotoxic at concentrations of 100 μg/mL. In addition, regarding the biodistribution of iron oxide nanoparticles in general, it has been reported that it depends on various parameters like the physiological barriers that they encounter, the administration route as well as their capabilities of crossing (or not) the barriers that they encounter [4,75,76,77,78]. Studies have shown that normally the liver and spleen are the most important organs involved in the process of eliminating the nanoparticles from the bloodstream [79,80,81,82]. Nonetheless, it was also reported that, when intravenously injected in high dosages, the liver and spleen macrophages are able to eliminate only partially these nanoparticles from the bloodstream, and the excess tends to get accumulated in other macrophage-rich tissues such as the lungs and adipose tissue [80]. Usually, when intravenously injected, the iron oxide nanoparticles enter in the blood vessels using the renal hilum and are excreted first by the ureter, then via the urinary bladder [81,82]. However, due to parameters such as size, there are no reports that describe the presence of non-degraded iron oxide nanoparticles in the urine, which means that these nanoparticles undergo a degradation process before being eliminated [83]. Moreover, it has been reported that, in some cases, the coating molecules that detached from the surface of the iron oxide nanoparticles because of their weak bonding and could be eliminated through the kidneys [84,85]. Even though the iron oxide nanoparticles have been reported to accumulate mostly in liver and spleen [77,86,87], they can also accumulate in the liver after reaching saturation in the spleen [88]. Both Gu et al. [89] and Briley-Saebo et al. [90] reported that, in the liver, the iron oxide nanoparticles are phagocytized by the Kupffer cells. Kupffer cells have the ability to degrade and metabolize partly or fully iron oxide nanoparticle and transform them in free iron and/or ferritin, with the help of liver hepatocytes [89,90]. More than that, it has been reported that depending on very specific parameters like size, coating and the presence of a specific targeting compound, iron oxide nanoparticles had the ability to cross several physiological barriers like blood brain barrier [91], placental [92] or skin barrier [93]. More than that, studies have shown that nanoparticles having hydrodynamic sizes between 15 and 100 nm exhibit the longest circulation time in the bloodstream, this way having a greater chance of reaching organs and targets, like the brain, the arterial walls, the lymph nodes or tumors [12,94,95]. Ferumoxide and Ferumoxtran-10 which are both used as MRI agents have similar dextran coatings, but their hydrodynamic diameter is different. Ferumoxtran-10, which has a longer circulation time (human blood half-life between 24 and 36 h), has been reported having a d_H_ between 15 and 50 nm, while Ferumoxides, which have a rapid circulation with a human blood half between 3.9 and 8 min, have been reported as having a d_H_ ranging from 62 to 80 nm [96,97]. Nonetheless, it has been reported that the elimination of iron oxide nanoparticles from the bloodstream of the human body is strongly dependent mostly on the pore’s sizes of blood vessel’s epithelium [98]. The research conducted in the last two decades has made tremendous improvements in the development of iron oxide nanoparticles with various shapes, sizes, coating layers and functional groups. The surface energy and reactivity of the particles surface has been engineered in order to reduce cytotoxicity and to improve their bio-compatibility [99,100]. Despite all this progress, the U.S. Food and Drug Administration [101] approved only a few superparamagnetic iron oxide nanoparticles for clinical use. Therefore, there is still a critical need for the understanding of the potential risks involved with the use of iron oxide nanoparticles. Disregarding the disadvantages and the potential risks, currently, there is a need for further in vitro and in vivo studies to explore the feasibility of developing and using iron oxide nanoparticles in a delivery and ablative treatment capacity.

## 4. Conclusions

In this study, dextran coated iron oxide aqueous magnetic fluids were obtained by an adapted coprecipitation method. The morphology of the DIO-AMF was assessed by TEM. The TEM micrographs revealed that the particles were well-dispersed with a narrow size distribution and spherical shape. The dextran coated iron oxide aqueous magnetic fluids showed a very good dispersion as it could be observed from the TEM and DLS studies. Moreover, a very good stability was evaluated by ultrasound measurements. A major contribution to the very good stability of the analyzed sample was due to the presence of dextran on the surface of magnetic nanoparticles. The diffraction characteristic peaks from the XRD patterns highlighted that the DIO-AMF particles are those of the cubic structure (Fd3m3 space group) of maghemite. The cytotoxicity assays evidenced that the DIO-AMF presented a small toxic effect at concentrations of 500 μg/mL for all the tested time intervals. The result obtained from the hematology investigations revealed that no effects were observed for the rats exposed to 62.5 μg/mL and 125 μg/mL doses containing DIO-AMF related to the control after 28 days. The concentration of 250 μg/mL of DIO-AMF influenced only the value of the test for the number of leukocytes, which shows a reactivity of the body manifested by an increase in the number of defense cells. The results presented in this paper indicate the importance of understanding both the in vitro and in vivo interactions of magnetic particles with cells and living organisms.

In concordance with in vitro research and in vivo toxicity examination, these results revealed that dextran coated iron oxide aqueous magnetic fluids at concentrations lower than 250 μg/mL have been shown to be safe. In conclusion, it can be said that these magnetic fluids could be used in environmental applications, such as water purification or in the medical and pharmaceutical field due to their low cytotoxicity.

## Data Availability

Data is available on demand from the corresponding author.
